# 3-Chloro-*N*-(4-hydr­oxy-3-methoxy­benz­yl)-2,2-dimethyl­propanamide

**DOI:** 10.1107/S1600536810009529

**Published:** 2010-03-20

**Authors:** Yan-Lan Huang, Wen-Long Wang, Shang Shan

**Affiliations:** a College of Chemical Engineering and Materials Science, Zhejiang University of Technology, People’s Republic of China

## Abstract

In the mol­ecular structure of the title compound, C_13_H_18_ClNO_3_, the amide group is nearly perpendicular to the benzene ring, making a dihedral angle of 85.66 (9)°. The C=O bond distance of 1.242 (3) Å and the C—N bond distance of 1.333 (3) Å suggest electron delocalization in the amide fragment. Inter­molecular O—H⋯O and N—H⋯O hydrogen bonding helps to stabilize the crystal structure.

## Related literature

The title compound is a derivative of capsaicin. For the biological activity of capsaicin, see: Kaga *et al.* (1989 ▶). For a related structure, see: Xia *et al.* (2009 ▶).
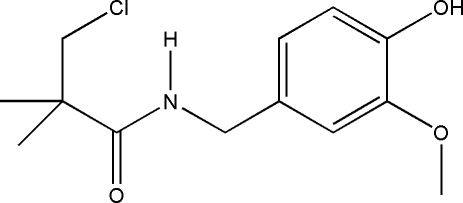


## Experimental

### 

#### Crystal data

C_13_H_18_ClNO_3_
*M* *_r_* = 271.73
Monoclinic, 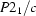

*a* = 9.3074 (10) Å
*b* = 11.5585 (13) Å
*c* = 13.0652 (14) Å
β = 90.378 (4)°
*V* = 1405.5 (3) Å^3^
*Z* = 4
Mo *K*α radiation
μ = 0.27 mm^−1^
*T* = 294 K
0.40 × 0.38 × 0.32 mm

#### Data collection

Rigaku R-AXIS RAPID IP diffractometer
15383 measured reflections
2732 independent reflections
2254 reflections with *I* > 2σ(*I*)
*R*_int_ = 0.042

#### Refinement

*R*[*F* ^2^ > 2σ(*F* ^2^)] = 0.060
*wR*(*F* ^2^) = 0.167
*S* = 1.05
2732 reflections
167 parameters
H-atom parameters constrained
Δρ_max_ = 0.48 e Å^−3^
Δρ_min_ = −0.67 e Å^−3^

### 

Data collection: *PROCESS-AUTO* (Rigaku, 1998 ▶); cell refinement: *PROCESS-AUTO*; data reduction: *CrystalStructure* (Rigaku/MSC, 2002 ▶); program(s) used to solve structure: *SIR92* (Altomare *et al.*, 1993 ▶); program(s) used to refine structure: *SHELXL97* (Sheldrick, 2008 ▶); molecular graphics: *ORTEP-3 for Windows* (Farrugia, 1997 ▶); software used to prepare material for publication: *WinGX* (Farrugia, 1999 ▶).

## Supplementary Material

Crystal structure: contains datablocks I, global. DOI: 10.1107/S1600536810009529/xu2733sup1.cif

Structure factors: contains datablocks I. DOI: 10.1107/S1600536810009529/xu2733Isup2.hkl

Additional supplementary materials:  crystallographic information; 3D view; checkCIF report

## Figures and Tables

**Table 1:** Hydrogen-bond geometry (Å, °)

*D*—H⋯*A*	*D*—H	H⋯*A*	*D*⋯*A*	*D*—H⋯*A*
O1—H1*A*⋯O3^i^	0.93	1.76	2.685 (2)	175
N1—H1*N*⋯O2^ii^	0.92	2.25	3.093 (3)	152

Symmetry codes: (i) 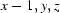; (ii) 
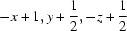
.

**Fig. 1.**
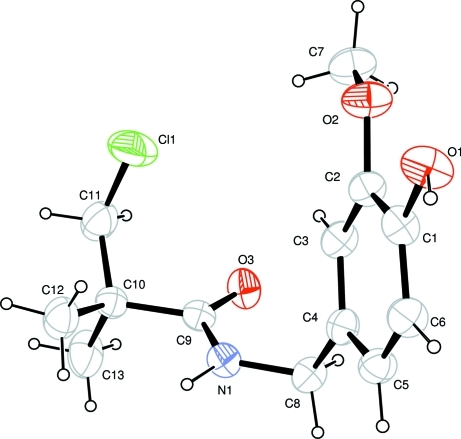
The molecular structure of the title compound with 30% probability displacement (arbitrary spheres for H atoms).

## supplementary crystallographic information

### Comment

Capsaicin, a pungent principle of capsicums, has been shown a variety of biological activities including mutagenicity (Kaga *et al.* 1989). During the investigation on syntheses of capsaicin derivatives, the title compound has recently been prepared in the labotory and its crystal structure is reported here.

The molecular structure of the title compound is shown in Fig. 1. The amide fragment is nearly perpendicular to the benzene ring [dihedral angle 85.66 (9))°]. The longer C9═O3 bond distance of 1.242 (3) Å and the shorter C9—N1 bond distance of 1.333 (3) Å suggest the electron delocalization in the amide fragment, which is comparable to that found in the related compound *N*-(4-Hydroxy-3-methoxybenzyl)benzamide (Xia *et al.* 2009).

Intermolecular O—H···O and N—H···O hydrogen bonding is present in the crystal structure (Table 1), which helps to stabilize the crystal structure.

### Experimental

4-Hydroxy-3-methoxy benzylamine HCl salt (4.7 g, 25 mmol) and dimethylformamide (25 ml) were added to a 100 ml 3-necked flask equipped with an additional funnel, a thermometer and a magnetic stirrer. Water solution (10 ml) of NaOH (2.0 g) was added at room temperature. The mixture was stirred at 308 K for 30 min and then cooled to 273 K. An ether solution (10 ml) of 2,2-dimethyl-3-chloropropionyl chloride (3.9 g, 25 mmol) was added dropwise at about 273 K over 15 min. After stirred for 2 h at room temperature the mixture was poured into 1M HCl solution (120 ml) , and then extracted with ethyl acetate. The ethyl acetate extract was washed with saturated NaHCO_3_ and brine. The extract was then dried over anhydrous Na_2_SO_4_ and filtered. Solvents were removed under vacuum at about 308 K to give a solid crude. Recrystallization was performed twice with an absolute ethyl acetate to obtain colourless single crystals of the title compound.

### Refinement

Hydroxy and imino H atoms were located in a difference Fourier map and were refined as riding in as-found relative positions with U_iso_(H) = 1.5U_eq_(N,*O*). Methyl H atoms were placed in calculated positions with C—H = 0.96 Å and torsion angle was refined to fit the electron density, U_iso_(H) = 1.5U_eq_(C). Other H atoms were placed in calculated positions with C—H = 0.93 (aromatic) and 0.97 Å (methylene), and refined in riding mode with U_iso_(H) = 1.2U_eq_(C).

### Crystal data

**Table:**

C_13_H_18_ClNO_3_	*F*(000) = 576
*M**_r_* = 271.73	*D*_x_ = 1.284 Mg m^−^^3^
Monoclinic, *P*2_1_/*c*	Mo *K*α radiation, λ = 0.71073 Å
Hall symbol: -P 2ybc	Cell parameters from 3466 reflections
*a* = 9.3074 (10) Å	θ = 2.2–24.0°
*b* = 11.5585 (13) Å	µ = 0.27 mm^−^^1^
*c* = 13.0652 (14) Å	*T* = 294 K
β = 90.378 (4)°	Prism, colorless
*V* = 1405.5 (3) Å^3^	0.40 × 0.38 × 0.32 mm
*Z* = 4	

### Data collection

**Table:**

Rigaku R-AXIS RAPID IP diffractometer	2254 reflections with *I* > 2σ(*I*)
Radiation source: fine-focus sealed tube	*R*_int_ = 0.042
graphite	θ_max_ = 26.0°, θ_min_ = 2.2°
Detector resolution: 10.0 pixels mm^-1^	*h* = −10→11
ω scans	*k* = −13→14
15383 measured reflections	*l* = −16→16
2732 independent reflections	

### Refinement

**Table:**

Refinement on *F*^2^	Secondary atom site location: difference Fourier map
Least-squares matrix: full	Hydrogen site location: inferred from neighbouring sites
*R*[*F*^2^ > 2σ(*F*^2^)] = 0.060	H-atom parameters constrained
*wR*(*F*^2^) = 0.167	*w* = 1/[σ^2^(*F*_o_^2^) + (0.0726*P*)^2^ + 1.051*P*] where *P* = (*F*_o_^2^ + 2*F*_c_^2^)/3
*S* = 1.05	(Δ/σ)_max_ = 0.002
2732 reflections	Δρ_max_ = 0.48 e Å^−^^3^
167 parameters	Δρ_min_ = −0.67 e Å^−^^3^
0 restraints	Extinction correction: *SHELXL97* (Sheldrick, 2008), Fc^*^=kFc[1+0.001xFc^2^λ^3^/sin(2θ)]^-1/4^
Primary atom site location: structure-invariant direct methods	Extinction coefficient: 0.021 (3)

### Special details

**Table:**

Geometry. All esds (except the esd in the dihedral angle between two l.s. planes) are estimated using the full covariance matrix. The cell esds are taken into account individually in the estimation of esds in distances, angles and torsion angles; correlations between esds in cell parameters are only used when they are defined by crystal symmetry. An approximate (isotropic) treatment of cell esds is used for estimating esds involving l.s. planes.
Refinement. Refinement of *F*^2^ against ALL reflections. The weighted *R*-factor wR and goodness of fit *S* are based on *F*^2^, conventional *R*-factors *R* are based on *F*, with *F* set to zero for negative *F*^2^. The threshold expression of *F*^2^ > σ(*F*^2^) is used only for calculating *R*-factors(gt) etc. and is not relevant to the choice of reflections for refinement. *R*-factors based on *F*^2^ are statistically about twice as large as those based on *F*, and *R*- factors based on ALL data will be even larger.

### Fractional atomic coordinates and isotropic or equivalent isotropic displacement parameters (Å^2^)

**Table:**

	*x*	*y*	*z*	*U*_iso_*/*U*_eq_	
Cl1	0.85692 (18)	0.09530 (8)	0.35770 (8)	0.1195 (6)	
N1	0.7804 (2)	0.39529 (17)	0.22613 (15)	0.0410 (5)	
H1N	0.7416	0.4356	0.2801	0.061*	
O1	0.18986 (18)	0.14975 (16)	0.13257 (15)	0.0539 (5)	
H1A	0.1120	0.1961	0.1482	0.081*	
O2	0.43484 (18)	0.03920 (15)	0.14718 (15)	0.0542 (5)	
O3	0.95555 (18)	0.27805 (16)	0.17024 (13)	0.0509 (5)	
C1	0.3123 (2)	0.2148 (2)	0.12573 (18)	0.0409 (5)	
C2	0.4447 (2)	0.1577 (2)	0.13548 (18)	0.0404 (5)	
C3	0.5709 (2)	0.2199 (2)	0.13426 (18)	0.0431 (6)	
H3	0.6581	0.1815	0.1422	0.052*	
C4	0.5700 (2)	0.3402 (2)	0.12130 (17)	0.0400 (5)	
C5	0.4394 (3)	0.3947 (2)	0.10673 (19)	0.0445 (6)	
H5	0.4371	0.4741	0.0953	0.053*	
C6	0.3112 (2)	0.3327 (2)	0.10891 (19)	0.0451 (6)	
H6	0.2243	0.3710	0.0990	0.054*	
C7	0.5646 (3)	−0.0248 (2)	0.1367 (2)	0.0572 (7)	
H7A	0.6070	−0.0079	0.0715	0.086*	
H7B	0.5441	−0.1061	0.1409	0.086*	
H7C	0.6303	−0.0036	0.1904	0.086*	
C8	0.7093 (3)	0.4073 (2)	0.12661 (18)	0.0445 (6)	
H8A	0.7732	0.3799	0.0735	0.053*	
H8B	0.6898	0.4885	0.1138	0.053*	
C9	0.8956 (2)	0.32899 (19)	0.24198 (17)	0.0371 (5)	
C10	0.9530 (2)	0.3193 (2)	0.35204 (17)	0.0410 (5)	
C11	1.0014 (4)	0.1958 (3)	0.3707 (2)	0.0669 (9)	
H11A	1.0419	0.1898	0.4391	0.080*	
H11B	1.0761	0.1761	0.3223	0.080*	
C12	0.8444 (3)	0.3548 (3)	0.4336 (2)	0.0599 (7)	
H12A	0.7575	0.3111	0.4245	0.090*	
H12B	0.8236	0.4358	0.4270	0.090*	
H12C	0.8838	0.3398	0.5003	0.090*	
C13	1.0859 (4)	0.3961 (3)	0.3605 (2)	0.0779 (11)	
H13A	1.1292	0.3865	0.4268	0.117*	
H13B	1.0586	0.4755	0.3513	0.117*	
H13C	1.1535	0.3746	0.3086	0.117*	

### Atomic displacement parameters (Å^2^)

**Table:**

	*U*^11^	*U*^22^	*U*^33^	*U*^12^	*U*^13^	*U*^23^
Cl1	0.2206 (15)	0.0570 (6)	0.0811 (7)	−0.0548 (7)	0.0203 (7)	−0.0059 (4)
N1	0.0349 (10)	0.0439 (11)	0.0441 (11)	0.0031 (8)	0.0016 (8)	−0.0048 (8)
O1	0.0314 (9)	0.0475 (10)	0.0829 (13)	0.0019 (7)	0.0038 (8)	−0.0097 (9)
O2	0.0373 (9)	0.0415 (10)	0.0838 (13)	0.0063 (7)	0.0059 (8)	0.0056 (9)
O3	0.0412 (10)	0.0648 (12)	0.0466 (10)	0.0112 (8)	0.0010 (7)	−0.0106 (8)
C1	0.0306 (12)	0.0464 (13)	0.0457 (12)	0.0014 (9)	0.0012 (9)	−0.0064 (10)
C2	0.0361 (12)	0.0403 (12)	0.0447 (12)	0.0060 (9)	0.0018 (9)	0.0004 (10)
C3	0.0334 (12)	0.0479 (14)	0.0480 (13)	0.0078 (10)	0.0009 (10)	0.0037 (10)
C4	0.0360 (12)	0.0466 (13)	0.0374 (11)	0.0020 (10)	−0.0009 (9)	0.0024 (9)
C5	0.0426 (14)	0.0401 (13)	0.0508 (14)	0.0043 (10)	−0.0033 (11)	0.0014 (10)
C6	0.0323 (12)	0.0461 (14)	0.0568 (14)	0.0087 (10)	−0.0050 (10)	−0.0034 (11)
C7	0.0478 (15)	0.0458 (15)	0.0780 (19)	0.0146 (12)	0.0061 (13)	0.0059 (13)
C8	0.0395 (13)	0.0484 (14)	0.0455 (13)	−0.0005 (10)	0.0000 (10)	0.0066 (10)
C9	0.0287 (11)	0.0374 (11)	0.0451 (12)	−0.0047 (9)	0.0023 (9)	−0.0013 (9)
C10	0.0382 (13)	0.0419 (13)	0.0429 (12)	−0.0041 (10)	−0.0008 (9)	−0.0005 (10)
C11	0.086 (2)	0.0616 (18)	0.0531 (16)	0.0212 (16)	−0.0002 (15)	0.0045 (13)
C12	0.0694 (19)	0.0669 (18)	0.0436 (14)	0.0124 (15)	0.0050 (12)	−0.0004 (12)
C13	0.072 (2)	0.102 (3)	0.0593 (18)	−0.0448 (19)	−0.0166 (15)	0.0090 (17)

### Geometric parameters (Å, °)

**Table:**

Cl1—C11	1.784 (4)	C6—H6	0.9300
N1—C9	1.333 (3)	C7—H7A	0.9600
N1—C8	1.462 (3)	C7—H7B	0.9600
N1—H1N	0.9206	C7—H7C	0.9600
O1—C1	1.369 (3)	C8—H8A	0.9700
O1—H1A	0.9252	C8—H8B	0.9700
O2—C2	1.382 (3)	C9—C10	1.535 (3)
O2—C7	1.424 (3)	C10—C11	1.516 (4)
O3—C9	1.242 (3)	C10—C13	1.526 (4)
C1—C6	1.380 (4)	C10—C12	1.530 (3)
C1—C2	1.403 (3)	C11—H11A	0.9700
C2—C3	1.378 (3)	C11—H11B	0.9700
C3—C4	1.400 (3)	C12—H12A	0.9600
C3—H3	0.9300	C12—H12B	0.9600
C4—C5	1.381 (3)	C12—H12C	0.9600
C4—C8	1.512 (3)	C13—H13A	0.9600
C5—C6	1.392 (3)	C13—H13B	0.9600
C5—H5	0.9300	C13—H13C	0.9600
			
C9—N1—C8	123.50 (19)	C4—C8—H8A	109.2
C9—N1—H1N	119.4	N1—C8—H8B	109.2
C8—N1—H1N	117.1	C4—C8—H8B	109.2
C1—O1—H1A	110.5	H8A—C8—H8B	107.9
C2—O2—C7	116.58 (19)	O3—C9—N1	121.3 (2)
O1—C1—C6	123.2 (2)	O3—C9—C10	121.1 (2)
O1—C1—C2	117.8 (2)	N1—C9—C10	117.55 (19)
C6—C1—C2	119.0 (2)	C11—C10—C13	107.3 (3)
C3—C2—O2	125.2 (2)	C11—C10—C12	109.7 (2)
C3—C2—C1	120.2 (2)	C13—C10—C12	109.5 (2)
O2—C2—C1	114.7 (2)	C11—C10—C9	108.7 (2)
C2—C3—C4	121.0 (2)	C13—C10—C9	107.60 (19)
C2—C3—H3	119.5	C12—C10—C9	113.9 (2)
C4—C3—H3	119.5	C10—C11—Cl1	112.0 (2)
C5—C4—C3	118.3 (2)	C10—C11—H11A	109.2
C5—C4—C8	121.7 (2)	Cl1—C11—H11A	109.2
C3—C4—C8	119.9 (2)	C10—C11—H11B	109.2
C4—C5—C6	121.1 (2)	Cl1—C11—H11B	109.2
C4—C5—H5	119.5	H11A—C11—H11B	107.9
C6—C5—H5	119.5	C10—C12—H12A	109.5
C1—C6—C5	120.4 (2)	C10—C12—H12B	109.5
C1—C6—H6	119.8	H12A—C12—H12B	109.5
C5—C6—H6	119.8	C10—C12—H12C	109.5
O2—C7—H7A	109.5	H12A—C12—H12C	109.5
O2—C7—H7B	109.5	H12B—C12—H12C	109.5
H7A—C7—H7B	109.5	C10—C13—H13A	109.5
O2—C7—H7C	109.5	C10—C13—H13B	109.5
H7A—C7—H7C	109.5	H13A—C13—H13B	109.5
H7B—C7—H7C	109.5	C10—C13—H13C	109.5
N1—C8—C4	112.03 (19)	H13A—C13—H13C	109.5
N1—C8—H8A	109.2	H13B—C13—H13C	109.5

### Hydrogen-bond geometry (Å, °)

**Table:**

*D*—H···*A*	*D*—H	H···*A*	*D*···*A*	*D*—H···*A*
O1—H1A···O3^i^	0.93	1.76	2.685 (2)	175
N1—H1N···O2^ii^	0.92	2.25	3.093 (3)	152

Symmetry codes: (i) *x*−1, *y*, *z*; (ii) −*x*+1, *y*+1/2, −*z*+1/2.
